# Supplementary material for: MetaRibo-Seq measures translation in microbiomes
Source: Nat Commun. 2020 Jun 29;11:3268. doi: 10.1038/s41467-020-17081-z (PMC7324362; doi:10.1038/s41467-020-17081-z)
Supplement: Supplementary file 10 — Supplementary Data 7 [file 41467_2020_17081_MOESM10_ESM.zip › File2/Confidence_High_Taxonomy/367942_out.krona.html]

Javascript must be enabled to view this page.

members
magnitude
magnitudeUnassigned
count
unassigned
taxon
rank

367942\_out

8

8
2
superkingdom

8
phylum
1224

class
8
1236

8
order
91347

8
family
543

8
genus
561

8
562
species

SRS012273\_contig\_number\_20275SRS049402\_contig\_number\_14334SRS064276\_contig\_number\_37578SRS076876\_contig\_number\_293SRS077127\_contig\_number\_11287SRS144362\_contig\_number\_39181SRS146888\_contig\_number\_15109SRS147022\_contig\_number\_14095
